# Bioprosthetic Aortic Valve Thrombosis and Literature Review

**DOI:** 10.3390/jcdd9080252

**Published:** 2022-08-06

**Authors:** Milan Radovanovic, Charles W. Nordstrom, Richard D. Hanna

**Affiliations:** 1 Mayo Clinic Alix School of Medicine, Rochester, MN 55905, USA; 2 Department of Hospital Medicine, Mayo Clinic Health System, Eau Claire, WI 54703, USA; 3 Department of Cardiology, Mayo Clinic Health System, Eau Claire, WI 54703, USA

**Keywords:** bioprosthetic aortic valve, thrombosis, elevated transvalvular gradients

## Abstract

An 83-year-old gentleman with a history of 23-mm Hancock-II-bioprosthetic aortic valve (BAV) replacement ten-years prior presented with symptoms of dyspnea and lower extremity edema. During the preceding seven-years, he had been noted to have asymptomatic increased mean transvalvular gradients (MG; 36–50 mmHg) felt to be due to either early bioprosthetic degeneration, pannus formation, or patient–prosthesis mismatch. An echocardiogram at the time of symptom development demonstrated significant flow acceleration through the aortic valve, mild regurgitation, and severely increased MG (48 mmHg) with prolonged acceleration time (AT, 140 msec). A trial of warfarin anticoagulation resulted in dramatic improvement after only 6 weeks with laminar flow through the AV, near-total resolution of regurgitation, and a decrease in MG to 14 mmHg and AT to 114 msec. These findings strongly suggest that BAV thrombosis was the predominant mechanism responsible for the longstanding high MG. Our case highlights that BAV thrombosis should be considered in the differential of elevated gradients regardless of the age of prosthesis, and that a trial of warfarin anticoagulation may be beneficial even if elevated gradients have been present for a prolonged period. Valvular gradients are often abnormal long before a formal diagnosis; however, these may reverse quickly with anticoagulation therapy.

## 1. Introduction

Bioprosthetic aortic valve (BAV) thrombosis is an increasingly recognized complication of tissue valve prosthesis, and it is considered one of the major mechanisms responsible for valve dysfunction in addition to BAV degeneration, pannus formation, or patient–prosthesis mismatch [1,2]. Although once considered rare, discrepancies between the doppler and catheter gradients of aortic valve prosthesis have been reported since the early 1990s [3]. Chronically elevated valvular gradients are hallmark findings and are often present long before symptom development and formal diagnosis [4,5]. As such, BAV thrombosis may be categorized as either subclinical or clinical [6].

Subclinical thrombosis in bioprosthetic surgical aortic valves (SAVI) appears to occur significantly less frequently and later when compared with transcatheter valves (TAVI) [5,7]. Furthermore, subclinical BAV thrombosis was historically deemed to occur primarily within 12 months following the implantation [5,7,8]. Subsequent observations have shown that BAV thrombosis may occur years following the prosthesis implantation [9]. Here, we present a unique case of therapeutically confirmed BAV thrombosis ten years following implantation and with seven years of consistently elevated gradients, and with aortic valve (AV) hemodynamic parameters normalizing following a 6-week treatment course of vitamin-K antagonist (VKA).

## 2. Case Description

An 83-year-old Caucasian gentleman with a history of hypertension and hyperlipidemia underwent SAVI with 23-mm Hancock II BAV due to severe aortic stenosis and aorta to right coronary artery bypass grafting in May of 2004 at the age of 73 years. Mid-2007, three years post-procedure, the patient was noted to have increased mean transvalvular gradients (MG) ranging from 36 to 50 mmHg with increased velocity (Table 1; post-operative and prior to 2007, echocardiographic studies were unavailable). Since the patient’s left ventricular (LV) systolic function remained within normal limits and he remained asymptomatic (completing farm chores without exercise limitations), his prosthetic parameters were not further investigated. Instead, he was followed by serial transthoracic echocardiograms (TTE) with a working diagnosis of bioprosthetic degeneration versus pannus formation or patient–prosthesis mismatch. The entire time patient remained on aspirin, 81 mg daily.

**Figure 1:**
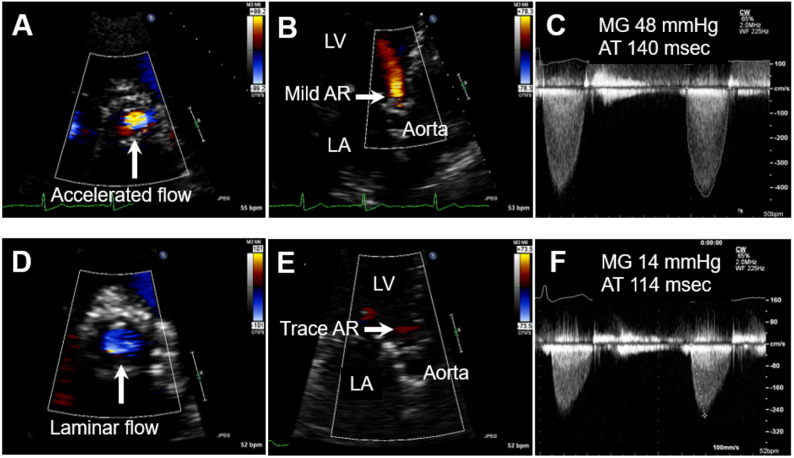
Transthoracic echocardiogram with CW Doppler before (**A**–**C**) and after (**D**–**F**) warfarin course (note virtually identical CW Doppler settings). (**A**) Short axis view demonstrating significant flow acceleration through the aortic prosthesis; (**B**) apical-axis view demonstrating mild aortic regurgitation (LV: left ventricle, LA: left atrium); (**C**) severely increased mean transvalvular gradient (MG) of 48 mmHg with prolonged acceleration time (AT) 140 msec. (**D**) Laminar flow through the aortic valve; (**E**) near disappearance of aortic regurgitation; (**F**) decrease in MG to 14 mmHg and AT to 114 msec.

In the spring of 2014, he developed insidiously worsening exertional dyspnea and peripheral edema. His primary care physician initiated furosemide, 40 mg daily, and referred him to his cardiologist. At that time, his vital signs were pertinent for blood pressure of 150/60 mmHg, a pulse of 76 beats/min, and oxygen saturation greater than 96%. On physical exam, he had a 2/6 systolic ejection murmur loudest at the right upper sternal border, which radiated to the carotids bilaterally and jugular venous pressure estimated at 10 cm of water. TTE was performed at the cardiology clinic and demonstrated significant flow acceleration through the aortic prosthesis, mild aortic regurgitation, and severely increased MG (48 mmHg) with prolonged acceleration time (AT; 140 msec) on continuous wave (CW) Doppler (Figure 1A–C). At that time, the diagnosis of BAV thrombosis was entertained, and the patient was initiated on a trial of warfarin anticoagulation with a target international normalized ratio (INR) of 2.5. The patient was scheduled for transesophageal echocardiography (TEE), which was delayed for 6 weeks owing to the patient’s wish to complete his spring planting. During this delay he continued warfarin therapy. Six weeks later TTE and TEE were completed and demonstrated a dramatic improvement in the prosthetic hemodynamics (the follow-up TTE was performed by the same echocardiography machine and was read by the same cardiologist as the initial TTE). There was laminar flow through the aortic valve, near-total resolution of aortic regurgitation, and a decrease of MG to 14 mmHg and AT to 114 msec (Figure 1D–F). Velocity was improved as well at 2.7 m/s. The patient’s symptoms had resolved, and he returned to his farm chores without limitations. These findings strongly suggest the predominant mechanism responsible for the longstanding high prosthetic gradients was indeed BAV thrombosis. The patient was continued on warfarin long term and remained asymptomatic until his decease a year later.

## 3. Discussion

The diagnosis of BAV thrombosis is challenging. Rather than direct visualization of the thrombus, diagnosis is usually based on echocardiographic identification of subtle morphologic and hemodynamic changes such as: increased cusp thickness, abnormal (decreased) leaflet motion, and 50% increases in prosthesis MG and AT [10]. These criteria have a high sensitivity and specificity for BAV thrombosis when seen in addition to the recently proposed Mayo Clinic diagnostic algorithm of decreased Doppler velocity index (DVI) of <0.25, and a more than 20% decrease in the effective orifice area (EOA) from the baseline [4].

Despite TTE being traditionally relied upon for structural and hemodynamic monitoring following prosthetic valve implantation, Makkar et al., in their paper resulting from the RESOLVE registry, reported that amongst TAVI patients the subtly reduced leaflet motion and change in hemodynamic parameters (commonly caused by subclinical BAV thrombosis) are better diagnosed by multidetector cardiac CT (CCT) imaging and TEE, and may be missed by TTE [8]. The mild increase in valvular gradients seen in subclinical BAV thrombosis are often within the expected echocardiographic range for BAV, and therefore may not be discernable by TTE [6]. CCT is highly accurate in assessing reduced leaflet motion (RELM) and leaflet morphology (specifically thickening), which is often referenced as hypoattenuating leaflet thickening (HALT). The use of CCT to evaluate subclinical BAV thrombosis, however, is not recommended outside clinical studies due to an unjustified exposure to radiation and contrast without evidence of treatment benefit in such cases [6,8]. Diagnosis of BAV can be made by pathological examination of the explanted prosthesis but is typically utilized only in patients with valve failure requiring repeated replacement [2,11]. Although embolic complications rarely occur in subclinical BAV thrombosis, valve thrombosis should be considered in any patient with a prosthetic heart valve presenting with an embolic event [12,13]. Furthermore, guidelines recommend yearly TTE beginning 5 years following implantation in asymptomatic patients [12,13].

The mechanism and risk factors for BAV thrombosis are not entirely certain. Immobile leaflets immediately following an aortic bioprosthetic valve implantation are frequently underrecognized and appear to be associated with early BAV dysfunction and thrombosis [2]. Early immobile leaflets are presumed to generate turbulent flow eddies across the prosthesis leaflets causing increased shear stress and premature deterioration of the valve, as well facilitating clot formation that may further impair leaflet mobility [2,14]. Increase of valve gradients immediately following SAVI have also been shown to be a predictor of BAV thrombosis, whereas following TAVI commissural misalignment was a reported predictor in some studies [15,16]. The impact of subclinical BAV thrombosis on postprocedural outcomes, valve durability and function, as well as the risk of mortality and stroke remains uncertain [6]. Recently, prolonged subclinical BAV thrombosis with increased gradients has been recognized as a cause of valve failure years following implantation [5,9]. There are data supporting that elevated valvular gradients are present months before BAV thrombosis is diagnosed clinically, thus yearly echocardiographic surveillance and increased awareness could lead to earlier diagnosis and more effective therapy [5].

With the intention to further investigate the time between BAV implantation and valve thrombosis diagnosis (time to BAV thrombosis), we performed a comprehensive literature search of the Medline database (National Library of Medicine, Bethesda, MD, USA) via the PubMed search engine from the inception until 1 July 2022. We used the following search keywords (combination of MeSH and non-MeSH terms): “bioprosthetic valve thrombosis“ AND “aorta OR aortic”. This search yielded 228 articles. We reviewed all surgical and transcatheter BAV thrombosis case reports and series, including cases reported in both observational and cohort studies, clinical trials, and systematic reviews that were diagnosed at least 1 year following implantation. Furthermore, the reference list of identified articles was manually screened to identify additional cases that could be included in our analysis. Results of time from valve implantation to BAV thrombosis diagnosis were displayed as a range (months). In articles without precise BAV data amongst other valves or valvular complications (in some papers valve thrombosis was represented as one of the reasons for valve failure), the highest value was used and was marked by the star in Table 2.

We found that the majority of BAV thrombosis cases occurred within 6 years. There was one case of bioprosthetic valve thrombosis reported by Egbe et al. which occurred more than 9 years following implantation, although not the precise valve location [10]. Upon reflection, this report may well be our case given that it originated from the same institution. Hattori et al. reported a case of acute myocardial infarction caused by thrombus derived from a large aneurysm of the sinus of Valsalva and BAV 10 years following implantation [23]. The authors hypothesized that the severely dilated aneurysm of the sinus of Valsalva precipitated turbulent blood flow resulting in a hypercoagulable state, and the stent strut of the BAV contributed to thrombus formation. Basra et al. reported BAV thrombosis diagnosed by CCT in 32 patients 5 days to 130.9 months following TAVI or SAVI [32]. The mean and median times from implantation to diagnosis were 27.8 and 14.2 months, respectively; any detailed report about the patient diagnosed with BAV thrombosis nearly 11 years following implantation is lacking. Although our patient had documented elevated gradients 3 years following SAVI, BAV thrombosis was not diagnosed and therapeutically confirmed until 10 years following implantation. As such, our report is a unique case of late BAV thrombosis. Despite continuously elevated gradients for 7 years, our patient remained asymptomatic and complication free. Likewise, in many of the reviewed cases, increased valvular gradients could be retroactively found on TTE months prior to the BAV thrombosis being diagnosed. Our report also demonstrates that even when gradients have been elevated for several years, BAV thrombosis may resolve promptly with VKA

Recommendations for mitigation of BAV thrombosis have not been completely standardized. United States (US) and European guidelines highlight an increased risk of BAV thrombosis within the first 3 months following implantation [12,13]. In patients with other indications for anticoagulation, lifelong oral anticoagulation is recommended following bioprosthetic SAVI or TAVI [12]. In patients who are otherwise without an indication for anticoagulation, the optimal antithrombotic strategy following a BAV implantation remains controversial and without high-quality evidence [12]. The general recommendation following bioprosthetic SAVI is to consider low-dose aspirin or VKA for the first 3 months [12]. There are many studies that support early use of VKA to reduce the risk of thrombosis and embolic complications; however, there is also evidence of increased major bleeding with VKA use compared with low-dose aspirin without a reduction in the rate of death or thromboembolic events [12,55]. Until recently, following TAVI, both US and European guidelines recommended dual antiplatelet therapy (DAPT) for the first 3 to 6 months, followed by lifelong single antiplatelet therapy (SAPT); however, the 2021 European guidelines were updated to recommend only lifelong SAPT following TAVI [12,13]. 

With respect to BAV treatment, the 2021 ESC/EACTS guidelines recommend VKA and/or unfractionated heparin before considering re-intervention (class I, level C recommendation) [12]. Anticoagulation should also be considered in patients with leaflet thickening and reduced leaflet motion leading to elevated gradients, with anticoagulation continued at least until resolution (class IIa, level B recommendation) [12]. Previous studies concluded that anticoagulation with VKA should be considered the first-line therapy in hemodynamically stable patients, as it usually results in hemodynamic and clinical improvement with minimal risk [1,9]. In early subclinical BAV thrombosis, novel oral anticoagulants (NOACs) may be as effective as warfarin [7], but data are lacking for late thrombosis. DAPT was found by many studies to be suboptimal for both the prevention and treatment of BAV thrombosis [7,8]. Redo surgery or thrombolytic therapy are reserved for hemodynamically unstable patients requiring urgent treatment [1,12].

Increased awareness of this entity over the past decade, in addition to updated recommendations and diagnostic algorithms, have led to earlier diagnosis and initiation of appropriate treatment [4]. Despite successful medical therapy and restoration of valve hemodynamics, BAV thrombosis continues to be a risk factor for accelerated bioprosthetic valve failure and repeated BAV thrombosis may occur [1,9,11,26]. Therefore, indefinite anticoagulation should be considered after initial the treatment of BAV thrombosis [26].

## 4. Conclusions

Our case highlights that BAV thrombosis should be considered in the differential of elevated bioprosthetic gradients regardless of the prosthesis age, and that a trial of oral anticoagulation with VKA may be beneficial even if elevated gradients have been present for a prolonged period. Valvular gradients are often abnormal long before the diagnosis is established and may reverse quickly with anticoagulation therapy.

## 5. Limitations of the Study

Limitations of our study are inherent to the nature of this type of literature review and include selection bias as well as publication bias. An additional limitation of our literature review is that we have included only cases in the English language and ones that were published in journals that are indexed in the Medline database. Although these strict criteria were implemented to avoid low-quality case reports, we recognize that we might have missed some high-quality cases if they did not meet our pre-selection criteria. 

## Figures and Tables

**Table 1:** Echocardiographic aortic valve parameters over a 7-year period and following a 6-week warfarin course.

Timeline	09/2007	11/2009	05/2011	10/2012	11/2013	04/2014		06/2014
MG (mmHg)	43	37	50	43	46	48	6-week warfarin therapy	14
Velocity (m/s)	4.0	4.1	4.5	4.3	4.4	4.4		2.7
LVEF (%)	65	60	60	65	58	63		56
Acceleration time (msec)	-	-	-	113	139	140		114
Aortic valve area (cm^2^)	-	-	0.9	1	0.90	0.77		1.54
Cardiac output (l/min)	-	-	-	-	5.95	4.81		5.80
Cardiac index (l/min/m^2^)	-	-	-	-	2.73	2.24		2.71
RV systolic pressure (mmHg)	43	55	60	54	64	58		60

Legend: MG—Mean gradient; LVEF—Left ventricular ejection fraction; RV—Right ventricle.

**Table 2:** A literature search of bioprosthetic aortic valve thrombosis with the time of bioprosthetic aortic valve implantation to thrombosis diagnosis displayed as a range value or highest value.

Reference	Number of Cases	Type of Valve Replacement (SAVI vs. TAVI)	Time from Valve Replacement to BAV Thrombosis Diagnosis (months)
Nuis et al. (2022) [17]	8	TAVI	Up to 60 months *
Bing et al. (2022) [18]	3	TAVI and SAVI	14–75 months
Andrade et al. (2022) [19]	1	SAVI	36 months
Naser et al. (2022) [2]	2	SAVI	Up to 50 months *
Naser et al. (2021) [5]	32	TAVI and SAVI	12.4–65.9 months
Kambeitz and Kemp (2021) [20]	1	SAVI	24 months
Bartus et al. (2021) [21]	1	SAVI	Less than 60 months *
Leon et al. (2020) [22]	16	TAVI and SAVI	Beyond 24 months, but not précised
Hattori et al. (2020) [23]	1	SAVI	120 months
Landes et al. (2020) [24]	14	TAVI	Beyond 12 months, but not précised
Chacon-Portillo et al. (2020) [25]	1	SAVI	24 months
Petrescu et al. (2020) [26]	62	TAVI and SAVI	9–68 months
Abdel-Wahab et al. (2020) [27]	3	TAVI	Up to 60 months
Kealhofer et al. (2019) [28]	1	SAVI	24 months
Bamford et al. (2019) [29]	2	SAVI	72 and 84 months
Balakrishnan et al. (2019) [30]	1	SAVI	96 months
Leatherby et al. (2019) [31]	1	SAVI	24 months
Egbe et al. (2018) [10]	53	TAVI and SAVI	12–43 months * ^§^ (one case up to 9 years)
Basra et al. (2018) [32]	32	TAVI and SAVI	0.2–130.9 months
Franzone et al (2018) [33]	10	TAVI	1–17.2 months
Fan et al. (2018) [34]	3	TAVI and SAVI	4 to 78 months
O’Callaghan et al. (2018) [35]	1	SAVI	48 months
Chakravarty et al. (2017) [7]	106	TAVI and SAVI	1–14 months
Egbe et al. (2017) [9]	31	TAVI and SAVI	13–80 months * ^§^
Vollema et al. (2017) [36]	16	TAVI	Up to 36 months
Couture et al. (2017) [37]	1	TAVI	54 months
Dalen et al. (2017) [38]	31	SAVI	1–41 months
Jose et al. (2017) [39]	18	TAVI	Up to 36 months
Regazzoli et al. (2016) [40]	1	TAVI	36 months
Del Trigo et al. (2016) [41]	68	TAVI	Up to 35 months
Galaska et al. (2016) [42]	1	SAVI	15 months
Egbe et al. (2015) [11]	29	TAVI and SAVI	12–60 months * ^§^
Makkar et al. (2015) [8]	39	TAVI and SAVI	Up to 23 months
Pislaru et al. (2015) [1]	11	SAVI	1–47 months
Latib et al. (2015) [43]	26	TAVI	Up to 24 months
Jander et al. (2015) [44]	17	SAVI	Up to 21.1 months
Cremer et al. (2015) [45]	1	SAVI	36 months
Orbach et al. (2013) [46]	1	TAVI	21 months
Brown et al. (2012) [47]	8	SAVI	3.5–20.5 months
Jander et al. (2012) [48]	6	SAVI	8–14 months
Peeceeyen et al. (2012) [49]	2	SAVI	18 and 60 months
Achouh et al. (2011) [50]	1	SAVI	24 months
Ohnaka et al. (2010) [51]	1	SAVI	27 months
Nishida et al. (2009) [52]	1	SAVI	24 months
Juliard et al. (1993) [53]	1	SAVI	40 months
Collins et al. (1983) [54]	1	SAVI	29 months

* Articles without precise BAV data amongst other valves or valvular complications, the highest value was used and was marked by a star. ^§^ Authors disclosed they used overlapping patients in papers by Egbe et al.

## Abbreviations

AT	acceleration time
AV	aortic valve
BAV	bioprosthetic aortic valve
CCT	cardiac CT
CW	continuous wave
DAPT	dual antiplatelet therapy
DVI	Doppler velocity index
EACTS	European Association for Cardio-Thoracic Surgery
EF	ejection fraction
EOA	effective orifice area
ESC	European Society of Cardiology
HALT	hypoattenuating leaflet thickening
INR	international normalized ratio
LV	left ventricle
MG	mean gradient
NOAC	novel oral anticoagulants
RELM	reduced leaflet motion
SAPT	single antiplatelet therapy
SAVI	surgical aortic valve implantation
TAVI	transcatheter aortic valve implantation
TEE	transesophageal echocardiogram
TTE	transthoracic echocardiogram
VKA	vitamin-K antagonist
